# Pre-stress performance in an instrumental training predicts post-stress behavioral alterations in chronically stressed rats

**DOI:** 10.3389/fnbeh.2015.00119

**Published:** 2015-05-13

**Authors:** Yoshio Iguchi, Sakurako Kosugi, Ziqiao Lin, Hiromi Nishikawa, Yoshio Minabe, Shigenobu Toda

**Affiliations:** ^1^Department of Psychiatry and Neurobiology, Kanazawa University School of MedicineKanazawa, Japan; ^2^Research Center for Child Mental Development, Kanazawa UniversityKanazawa, Japan

**Keywords:** appetitive instrumental learning, chronic unpredictable stress, individual difference, major depressive disorder, pre-stress traits, post-stress phenotypes

## Abstract

Stress is a major factor in the development of major depressive disorder (MDD), but few studies have assessed individual risk based on pre-stress behavioral and cognitive traits. To address this issue, we employed appetitive instrumental lever pressing with a progressive ratio (PR) schedule to assess these traits in experimentally naïve Sprague-Dawley rats. Based on four distinct traits that were identified by hierarchical cluster analysis, the animals were classified into the corresponding four subgroups (Low Motivation, Quick Learner, Slow Learner, and Hypermotivation), and exposed to chronic unpredictable stress (CUS) before monitoring their post-stress responses for 4 weeks. The four subgroups represented the following distinct behavioral phenotypes after CUS: the Low Motivation subgroup demonstrated weight loss and a late-developing paradoxical enhancement in PR performance that may be related to inappropriate decision-making in human MDD. The Quick Learner subgroup exhibited a transient loss of motivation and the habituation of serum corticosterone (CORT) response to repeated stress. The Slow Learner subgroup displayed resistance to demotivation and a suppressed CORT response to acute stress. Finally, the Hypermotivation subgroup exhibited resistance to weight loss, habituated CORT response to an acute stress, and a long-lasting amotivation. Overall, we identified causal relationships between pre-stress traits in the performance of the instrumental training and post-stress phenotypes in each subgroup. In addition, many of the CUS-induced phenotypes in rats corresponded to or had putative relationships with representative symptoms in human MDD. We concluded that the consequences of stress may be predictable before stress exposure by determining the pre-stress behavioral or cognitive traits of each individual in rats.

## Introduction

Stress may lead to various aversive outcomes, and major depressive disorder (MDD) is one of the most damaging consequences (Collins et al., 2011). The increasing number of MDD patients is imposing a growing worldwide social and economical burden (World Health Organization, 2014), and some of these patients are resistant to first-line medications (Rush et al., 2006). Numerous preclinical studies have attempted to identify the molecular and cellular mechanisms that underlie MDD to develop effective therapies (McEwen, 2005; Russo and Nestler, 2013).

However, MDD prevention, diagnosis, or medication of MDD cannot be formulated or standardized for each case due to various individual differences. For example, the responses to stress vary substantially among individuals, including animals and humans. Some may exhibit resilience, whereas others may exhibit MDD-like vulnerability after the same stress exposure (Krishnan et al., 2007). In addition, the effectiveness of antidepressants in treating MDD varies among individuals (Trivedi et al., 2006), while the predominant clinical manifestations, severity, or courses differ markedly among individuals (Rush, 2007), which has prompted physicians to subtype this disorder (Harald and Gordon, 2012). It would be highly beneficial if we could predict the consequence for each individual before exposure to a stress that could trigger MDD. This identification could be useful given the individual differences in causality between individual pre-onset traits and post-onset phenotypes. This causality has been investigated vigorously in human longitudinal studies (Fuhr et al., 2014). The corresponding approach has also been applied to preclinical studies using animal models of other mental disorders, such as addiction (Belin et al., 2011; Saunders and Robinson, 2011; Broos et al., 2012), but very few animal studies in terms of MDD (Stedenfeld et al., 2011; Duclot and Kabbaj, 2013; Rygula et al., 2013).

To investigate the possible relationships between pre-stress traits and both early and late-emerging post-stress MDD-related behaviors in rats, we first measured the motivation and bias of action selection in cost- or effort-based decision-making using instrumental training with a progressive ratio (PR) schedule (Kurniawan et al., 2011; Der-Avakian and Markou, 2012) to establish pre-stress phenotypic subgroups. We then re-tested these subgroups for 4 weeks after chronic unpredictable stress (CUS) exposure to identify the causal relationship between pre-stress traits and post-CUS phenotypes.

## Materials and methods

### Animals

All animal procedures were in accordance with Guidelines for Proper Conduct of Animal Experiments (Science Council of Japan, June 2006) and approved by Kanazawa University IACUC. Male Sprague–Dawley rats (Japan SLC, Hamamatsu, Japan) initially weighing 275–300 g were housed 2 or 3/Plexiglas cage (38 × 33.5 × 17 cm) in a climate-controlled vivarium with a 12/12-h light-dark cycle (lights on at 08:45 h). Instrumental learning trainings (fixed- and progressive-ratio training, set-shifting, and response reversal learning) were conducted during the light phase.

### Apparatus

Instrumental learning was performed in operant chambers equipped with two retractable levers (30 × 24 × 25 cm; Med associates, St. Albans, VT, USA) in a soundproof room. Stimulus light (100 mA, 28 V) for visual cue discrimination learning was mounted above each lever. Chambers were also equipped with a miniature solenoid-activated valve to deliver 50 μl of 0.2% (w/v) sodium saccharin dihydrate (Wako Pure Chemical Industries, Osaka, Japan) into a recessed magazine between the two levers, except for set-shifting and response reversal learning in which the magazine was relocated to the wall opposite the levers. One 100 mA, 28 V houselight on the top center of the wall opposite the levers provided ambient illumination.

Cocaine- and novelty-induced behaviors were monitored using three photoelectric actimeters (Panlab, Barcelona, Spain), each comprising a novel environment (NE) of transparent acrylic plastic (45 × 45 × 30 cm) under faint illumination (approximately 0.5 lx). The floor was covered with wood chips. Two 16 × 16-infrared beam arrays located diagonally at the NE edge were used to monitor locomotor (horizontal) activity.

### Timelines of all experiments

**Figure 1:**
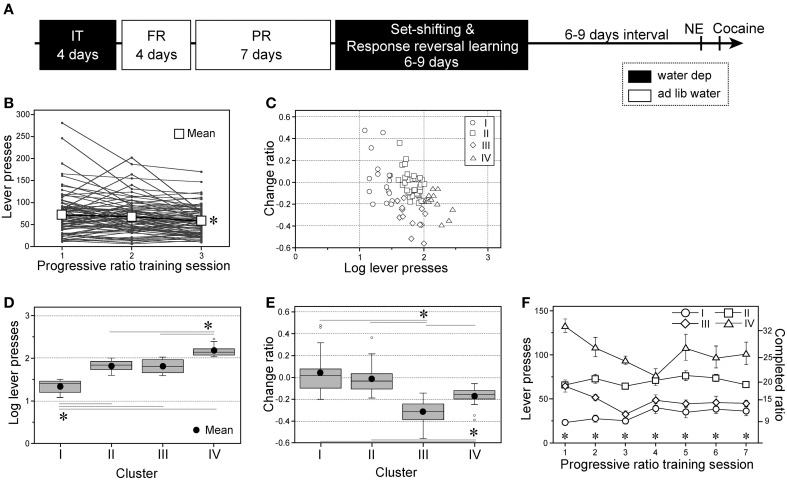
**Individual variability in progressive ratio instrumental performance of subgroups. (A)** A chronological schema of the entire experimental procedure of Experiment 1. IT, initial instrumental training; FR and PR, fixed- and progressive-ratio instrumental trainings, respectively; NE and cocaine, novel environment- and cocaine-induced behaviors testing, respectively. Black and white boxes = the days with or without water deprivation, respectively. **(B)** Individual performances with progressive ratio (PR) instrumental training for the first three training sessions. The data of the third session was compared with those of the first and second sessions [*session*: *F*_(2, 156)_ = 10.01, *p* < 0.0001] **(C)** Each rat's performance plotted on a two-factorial surface. *x*-axis: number of lever presses in the first PR session normalized by a common logarithm (log) transformation; *y*-axis: change ratios of lever presses from first (*a*) to third (*b*) sessions, (*b* − *a*)/(*b* + *a*). **(D,E)** Box plots of the log-transformed lever press numbers during the first PR session; Cluster I, *n* = 17; II, *n* = 36; III, *n* = 13; IV, *n* = 13 [**D**, *subgroup*: *F*_(3, 75)_ = 106.42, *p* < 0.0001] and the change ratio [**E**, *subgroup*: *F*_(3, 75)_ = 20.58, *p* < 0.0001]. **(F)** Active lever presses during seven PR sessions by four subgroups. Data are shown as subgroup mean ± SEM; Cluster I, *n* = 13; II, *n* = 32; III, *n* = 12; IV, *n* = 10 [*subgroup* × *session* interaction: *F*_(18, 376)_ = 3.52, *p* < 0.0001]. ^*^p < 0.05, between sessions **(B)**, between relevant subgroups **(D,E)**, or significant simple-main effects of *subgroup* on each session **(F)**.

In Experiment 1, following 4-day initial training, rats were subjected to the trainings of fixed- (FR; 4 days) and progressive-ratio (PR; 7 days) instrumental learning. Then, performance in set-shifting and response reversal learning was examined. 6–9 days later, cocaine-induced behaviors were assessed in a NE. In Experiment 2, animals were exposed to chronic unpredictable stress (CUS) or daily handling as control treatment for 28 days following FR and PR instrumental training. Then, animals were maintained for 28 days (post-CUS period) with weekly behavioral assays. A chronological schema of the entire experimental procedure of Experiment 1 and 2 are depicted in Figures 1A, 3A, respectively.

### Time-constraint progressive ratio instrumental training

Experimentally naïve rat groups (Experiment 1: *n* = 79; Experiment 2: *n* = 84) handled daily for 5 days before training were presented with bottles of saccharin solution in their home cages for 24 h before training to ameliorate neophobia to an unfamiliar reward. Response to one lever was rewarded (active lever), whereas that to the other lever was not (inactive lever). Active and inactive lever positions were counterbalanced across animals. First, rats received a 30-min training session over 4 successive days for apparatus habituation, magazine training, manual shaping, and continuous reinforcement training with the active lever alone following 23.5-h water deprivation. After daily session completion, animals were allowed to drink water for 30 min in their home cages. Then, the water-restriction schedule was lifted (i.e., *ad libitum* access to water and food). Animals were then subjected to 4 training sessions (once/day) with both levers and changing lever press: reward ratios of 1:1 (FR-1, session 1), 2:1 (FR-2, session 2), and 5:1 (FR-5, sessions 3 and 4). Each session was terminated after 100 rewards or 30 min. Daily training sessions with a PR schedule for 7 (Experiment 1) or 3 days (Experiment 2) followed after them. The PR schedule was based on the following exponential progression: 1, 2, 4, 6, 9, 12, 15, 20, 25, 32, 40,…, derived from the formula [(5 × e^0.2^*^n^*) - 5] rounded to the nearest integer, where *n* is the trial number within a session (Richardson and Roberts, 1996). At the start of each daily session, active and inactive levers were inserted into the chamber after a 300-s waiting time, followed by a 35-min long session. Motivation was evaluated as the number of lever presses in each session (and the corresponding highest completed ratio) (Barr and Phillips, 1998).

### Set-shifting and response reversal learning

In Experiment 1, a 23.5-h/day water deprivation schedule was resumed after a 7-day PR training completion, and performance in set-shifting and response reversal learning was examined as described (Floresco et al., 2008; Haluk and Floresco, 2009) with minor modifications. On the day following the last PR session, the magazine was moved to the wall opposite the levers to reform the positional relationship. Animals were retrained on an FR-1 schedule to a criterion of 90 reinforcements in 30 min. After familiarization with insertion and retraction of levers, animals were trained to press them within 10-s insertion. A session comprised 90 trials at 20-s intervals. A trial began with houselight illumination and insertion of either lever into the chamber. Failure of the rat to press the lever within 10 s resulted in lever retraction and chamber darkening; the trial was scored as an omission. Responses by the rat within 10 s resulted in lever retraction, an immediate reward, and houselight illumination for another 4 s. Right and left levers were presented once in every pair of trials in a random order. Each animal had to achieve a criterion of 5 or fewer omissions over 90 trials to proceed to set-shifting the next day. In set-shifting, animals were first trained to press a lever with overhead cue light illumination (visual cue discrimination), then to press one particular lever (left or right) consistently while ignoring the visual cue (response discrimination). Retraction of both levers and chamber darkening occurred before each visual cue discrimination training session [=inter-trial interval condition (ITI)]. Every 20 s, a trial began with illumination of the stimulus cue light; 3 s later, the houselight was turned on, and both levers were inserted into the chamber. Responses to the correct lever (beneath the cue light) resulted in retraction of both levers, a reward, and houselight illumination for another 4 s, after which the chamber returned to ITI. Incorrect responses resulted in retraction of both levers and the houselight being turned off immediately without reinforcement. Failure to press either lever within 10 s was recorded as an omission; the chamber returned to ITI. For each animal, trials continued until a minimum of 30 trials and a criterion performance of 8 consecutive correct choices were achieved. On the following day, response discrimination training commenced. The ITI and criterion of success were identical to those in the visual cue discrimination task, within a maximum of 112 trials.

On the following day of set-shifting, reversal learning commenced. Animals were trained to press the lever resulting in a reward during the preceding set-shifting training (initial response discrimination); then, the position of the correct lever was reversed on the next day. For reversal learning, the ITI condition, houselight illumination, and success/failure criteria were the same as those for set-shifting, except that the cue light was not presented.

Errors made during set-shifting were categorized into subtypes, perseverative, regressive, and never-reinforced, described previously (Floresco et al., 2008; Haluk and Floresco, 2009). Perseverative errors were scored when a rat responded to the lever with a cue light in trials requiring response to the opposite lever. Eight of every 16 consecutive trials required responses in this manner, and these trials were separated into consecutive blocks of 8 trials each. Perseverative errors were scored when a rat pressed the incorrect lever in 6 or more of 8 trials/block. Once a rat made 5 or fewer perseverative errors in a block for the first time, all subsequent errors of this type were no longer counted as perseverative, but instead scored as regressive. Never-reinforced errors were scored when a rat pressed the incorrect lever in trials with cue light illumination above the correct (rewarded) lever. Regressive and never-reinforced errors were used as indices of maintaining and acquiring a new strategy, respectively, and perseverative errors as an index of disengagement from a previously relevant learning rule.

### Cocaine-induced behaviors

In Experiment 1, the water-restriction schedule was again lifted on set-shifting and response reversal learning completion; 6–9 days later, animals were placed into a NE for 1-h acclimation. On the next day, animals were placed into the NE for 30 min to monitor spontaneous activity, then injected intraperitoneally with 15 mg/kg cocaine hydrochloride (Takeda Pharmaceutical Co., Osaka, Japan), and placed back into the NE for 120 min to assess cocaine-induced behaviors.

### CUS exposure

In Experiment 2, naive rats were assigned to either the handled (control) or CUS-exposed group after completion of 3 daily PR sessions. The CUS group was housed singly with *ad libitum* access to food and water and subjected to CUS as described previously (Dias-Ferreira et al., 2009) with some modifications. During the 4-week period, rats were exposed to 8 cycles of 3 kinds of stressor, restraint, forced swimming, or social defeat (representing psychological, physical, and social stressors, respectively), with 4 stress-free days. One of the three stressors was imposed every day in an unpredictable order at various times during the light phase. Intensities of these stressors were escalated on a weekly basis to prevent habituation. In restraint stress sessions, each animal was immobilized inside a size-fitted, transparent polyethylene terephthalate tube for 30 min during the first week, 45 min during the second week with simultaneous illumination from a 200-W bulb plus 5-min rotary shaking, and 60 min during third and fourth weeks with illumination plus 20-min shaking. In forced swimming, each animal was placed in an opaque plastic bucket (57-cm tall, 43-cm diameter) filled with water (21 ± 2°C) to a 35-cm depth for 10 min during the first week, 15 min during the second, and 20 min during third and fourth weeks. On completion, animals were dried and returned to the home cage. Social defeat was based on the resident–intruder paradigm (Tornatzky and Miczek, 1994; Koolhaas et al., 1997). Ten male Long–Evans rats weighing around 150 g more than experimental subjects at the start of CUS were used as residents. To enhance territoriality, the resident was housed with a female Long–Evans rat until resident–intruder sessions. Some old bedding was always retained on cleaning residents' cages (weekly). The subject rat (intruder) was placed into the resident home cage, and they were allowed to interact until the intruder showed submissive supine posture or freezing or if 10 min had elapsed. During the first week, intruders were removed from the resident home cage and returned to their own home cage immediately following defeat (or 10 min). During the second–fourth weeks, the resident was physically separated from the intruder following defeat by a wire-mesh partition and forced to remain in proximity to the resident for 30 min (second week) or 60 min (third and fourth weeks). To reduce habituation induced by repeatedly encountering the same resident, intruders confronted different residents in each session. Animals assigned to the control group were housed in groups of two or three and handled daily during the CUS period.

### Post-CUS behavioral measurements

After CUS or control handling completion, all animals were housed in groups for 28 days (post-CUS period). On the day following the last CUS session, each animal was placed into the operant chamber and presented with active and inactive levers. They were retrained on an FR-1 schedule with the active lever to a criterion of 20 reinforcements in 30 min. This session intended to assess effects of CUS on low-cost instrumental performance and to reinstate previously trained appetitive instrumental behavior. Performance on the PR schedule was tested on post-CUS Days 2, 11, 19, and 27. Tests were conducted under conditions similar to those for 3 daily PR sessions before CUS with *ad libitum* access to either water or food. On post-CUS Days 3 and 28, animals were placed in a NE for 30 min. To preserve the novelty of the NE during repeated measurements, the configuration was changed before the second session (black dotted wall paper with sawdust bedding or black striped wall paper with paper-litter bedding, order counterbalanced across animals). Body weight was recorded weekly during both CUS and post-CUS periods.

### Serum corticosterone levels at baseline and after acute stress exposure

In Experiment 2, morning blood samples (between 9:00 and 13:00) were collected from the tail vein for the measurement of resting state corticosterone concentration. These samplings were conducted firstly 2 days before CUS onset, secondly on Day 27 of the CUS period (= early post-CUS), and lastly on Day 25 of the post-CUS period (= late post-CUS) as depicted in **Figure 3A**. On the day before CUS Day 1 and on post-CUS Day 26, rats were placed individually in an electric-shock chamber (40 × 15 × 40 cm) equipped with an electrified grid floor (each grid bar: 0.4-cm diameter, 1.6-cm apart) for acute stress response testing. After a 5-min acclimation period, animals received 5 unavoidable electric footshocks (40 V, 1 s) with, on average, a 2-min inter-shock interval. Blood was collected from the tail vein 15 min after the last shock. Supernatants (1000 × *g* for 15 min and then 2000 × *g* for 10 min, 4°C) were stored at −80°C until use. Corticosterone concentrations were determined using an ELISA kit (AssayPro, St. Charles, MO) according to the manufacturer's protocol.

### Statistical analyses

Hierarchical clustering to classify naïve animals based on PR instrumental performance (Experiment 1) was conducted using R ver. 2.15.3 software (R Core Team, 2012). Between-group differences were tested using analyses of variance (ANOVA) and *post hoc* multiple comparisons with Ryan's procedure (Ryan, 1960). Two-Way or Three-Way repeated-measures ANOVAs were also used to analyze within-group repeated measures. *Post hoc* comparisons were performed by using simple-main or simple-simple-main effects tests and multiple comparisons with Ryan's procedure. Data points ± 2*SD* from the subgroup mean were discarded in analysis of the set-shifting and reversal learning data. All statistical analyses were performed using ANOVA 4 on the Web (http://www.hju.ac.jp/~kiriki/anova4). Result reliability was assessed against a type I error (*α*) of 0.05 unless otherwise noted.

## Results

### Classification of individual pre-stress traits based instrumental performance

In Experiment 1 (Figure 1A), we trained a group of experimentally naïve rats to press an active lever on a time-constrained (35 min) PR schedule for 7 days. The mean number of lever presses declined progressively with each session, and significant differences were detected based on multiple comparisons between the first and third sessions, and between second and third sessions [*t*s_(156)_ = 4.38 and 2397, respectively; Figure 1B]. In particular, considerable individual variability was observed on the first day. Therefore, we aimed to classify the animals based on two factors: (1) lever presses in the first PR session normalized by a common logarithm (log) transformation and (2) the change in the number of lever presses from the first to third sessions [change ratio = (*b* − *a*)/(*b* + *a*), where *a* and *b* are the lever presses in the first and third sessions, respectively]. The performances of all animals fell within a relatively restricted area of a two-factorial graph (Figure 1C), and hierarchical clustering based on the Euclidean squared distances between data points defined four subgroups (Supplementary Figure 1): Cluster I, *n* = 17 (21.5% of all animals); Cluster II, *n* = 36 (45.6%); Cluster III, *n* = 13 (16.5%); and Cluster IV, *n* = 13 (16.5%). Cluster I performed significantly fewer lever presses in the first PR session than other three subgroups [*t*s_(75)_ ≥ 9.64], whereas Cluster IV performed significantly more lever presses than Clusters II and III (*t*s = 8.23 and 7.31, respectively: Figure 1D). Cluster III exhibited significantly lower change ratios than the other three subgroups [*t*s_(75)_ ≥ 2.56], whereas Cluster IV exhibited significantly lower change ratios than Clusters I and II (*t*s = 4.09 and 3.49, respectively; Figure 1E).

Next, we analyzed the subgroup differences in terms of their responses to active levers over seven PR sessions (Figure 1F; Cluster I, *n* = 13; Cluster II, *n* = 32; Cluster III, *n* = 12; Cluster IV, *n* = 10; the remaining animals were used for brain sampling, data not shown). In the third session, Cluster IV performed significantly more lever presses than the other three subgroups [*t*s_(441)_ ≥ 3.24] and Cluster II performed significantly more lever presses than Clusters I and III (*t*s = 4.85 and 3.75, respectively). Similarly, in the seventh session, Cluster IV performed significantly more lever presses than the other three subgroups (*t*s ≥ 3.89), and Cluster II performed significantly more lever presses than Clusters I and III (*t*s = 3.72 and 2.54, respectively). Similar subgroup differences were obtained for the inactive lever performance levels during the seven sessions (data not shown). In addition, we retrospectively analyzed subgroup differences in active lever presses during FR training, which preceded the PR training (Supplementary Figure 2). No significant subgroup differences were found during FR-1 training with water deprivation. However, significant subgroup differences emerged after water became accessible *ad libitum*. In the first session (FR-1) Cluster IV performed significantly more active lever presses than Clusters I and III [*t*s_(300)_ = 3.78 and 2.92, respectively] and Cluster II performed significantly more lever presses than Cluster I (*t* = 2.89). Almost the same results were obtained from the second (FR-2) to the last (FR-5) sessions. In the last session, Cluster IV performed significantly more active lever presses than any of the other three subgroups (*t*s ≥ 7.09) and Cluster I performed significantly fewer lever presses than Clusters II and III (*t*s = 4.41 and 2.99, respectively).

**Figure 2:**
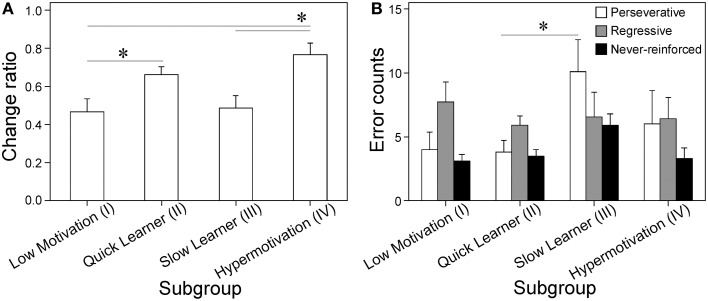
**Differential sensitivity to reward devaluation and variable number of errors during set-shifting of each subgroup. (A)** Change ratios of active lever response rates (lever presses/min) with the fixed ratio reward schedule (FR-1) with vs. without water deprivation (index of sensitivity to devaluating the fluid reward); Cluster I, *n* = 17; II, *n* = 36; III, *n* = 13; IV, *n* = 13 [*subgroup*: *F*_(3, 75)_ = 4.87, *p* = 0.0038]. **(B)** Analyses of three error types (perseverative, regressive, and never-reinforced) made during set-shifting by four subgroups. Data are shown as subgroup mean + SEM (data located outside mean ± 2*SD* were discarded from the statistical analyses, changing the numbers of animals in each subgroups as follows; Cluster I, *n* = 11; II, *n* = 27; III, *n* = 11; IV, *n* = 7), [*subgroup* on perseverative error: *F*_(3, 52)_ = 3.04, *p* = 0.037; regressive error: *F*_(3, 52)_ < 1; never-reinforced error: *F*_(3, 52)_ = 3.02, *p* = 0.038]. ^*^p < 0.05, between relevant subgroups.

To identify the distinct behavioral and cognitive characteristics of the four subgroups, we calculated the individual change ratios in the response rates (lever presses/min) with the active lever between FR-1 training sessions with and without water deprivation (Supplementary Figure 2). This index (*ad libitum* water/with water deprivation) reflects the behavioral sensitivity to the change in the reward incentive value for a fluid reward (0.2% saccharin solution) because the incentive value was reduced by relieving water deprivation. As shown in Figure 2A, Cluster I had significantly lower change ratios than Clusters II and IV [*t*s_(75)_ = 2.59 and 3.18] and Cluster III had significantly lower change ratios than Cluster IV (*t* = 2.78).

Based on the differences in instrumental performance with the PR schedule, the subgroups were designated as follows. Clusters I was designated as “Low Motivation” and Cluster IV as “Hypermotivation,” because these subgroups performed the lowest and highest numbers of lever presses to obtain rewards during PR training, respectively. Cluster II was designated as “Quick Learner (QL)” because its members rapidly acquired a habitual action that was insensitive to changes in the reward value during PR training. Cluster III was designated as “Slow Learner (SL),” because its members still appeared to be sensitive to changes in the reward value compared with QL (Yin and Knowlton, 2006).

### Four subgroups exhibited distinct errors during set-shifting

We compared the performance in set-shifting and response reversal learning paradigms among the four subgroups. In set-shifting, the animals were first trained on a visual cue discrimination task where they chose the lever with an overhead light illumination cue. This training process progressed steadily without significant group differences (data not shown). In the subsequent set-shifting task, where the animals had to press the lever consistently while ignoring the visual cue, the SL subgroup made significantly more errors than the Low Motivation and QL subgroups [*t*s_(52)_ = 2.84 and 4.10, respectively; data not shown]. Analyses of error types showed that SL committed significantly more perseverative errors than QL (*t* = 2.90: Figure 2B). Significant effects of *Subgroup* were not found for regressive errors, whereas they were for never-reinforced errors (*p* < 0.05).

The animals were then trained by reversal learning, which first required the consistent choice of the previously rewarded (cued) lever (initial response discrimination) and then consistent choice of the other. This training also proceeded as expected without significant subgroup differences (data not shown). Similarly, None of subgroups were statistically significant in terms of the mean number of errors made during the subsequent response reversal learning as well (data not shown).

### Distinct behavioral responses of subgroups to a novel environment and acute cocaine administration

There were no significant subgroup differences in the distance traveled (novel environment, NE) before cocaine administration (Supplementary Figure 3A). However, because a *Subgroup* (4) × *Time-block* (24) ANOVA showed a marginal significant interaction between the factors [*F*(69, 874) = 1.27, *p* = 0.076], we conducted subsequent simple-main effect tests and multiple comparisons. The Hypermotivation subgroup traveled a significantly greater distance than any of the other three subgroups in the third time-block after acute cocaine administration [*t*s_(912)_ ≥ 3.21] and significantly greater distance than the QL subgroup in the second block (*t* = 2.89) and the Low Motivation subgroup in the fifth and seventh blocks (*t*s = 2.41 and 2.52, respectively; Supplementary Figure 3B).

Supplementary Table 1 summarizes all of the distinct pre-CUS characteristics of the four subgroups.

### Distinct subgroup responses in terms of peripheral corticosterone levels and body weight gain during and after CUS

**Figure 3:**
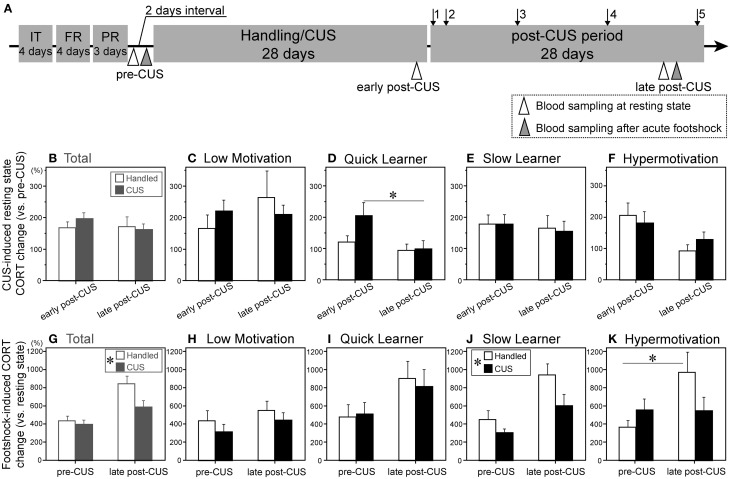
**Variable serum corticosterone levels in the resting state and following acute stress exposure in subgroups. (A)** A chronological schema of the entire experimental procedure of Experiment 2. IT, initial instrumental training; FR and PR, fixed- and progressive-ratio instrumental trainings, respectively; arrow 1, fixed rato-1 test; arrows 2–5, 4 progressive ratio tests conducted during the post-CUS period. Open and gray triangles = the day of morning blood sampling for resting state serum corticosterone (CORT) and of 15 min after footshock, respectively. **(B–F)** Change in CORT levels during resting state between early and late post-CUS timepoints, presented as % of pre-CUS levels, of the total cohort (Total, **B**) and each subgroup (**C–F**) (Low motivation [LM]-CUS, *n* = 15; LM-Handled, *n* = 12; Quick Learner [QL]-CUS, *n* = 6; QL-Handled, *n* = 7; Slow Learner [SL]-CUS, *n* = 17; SL-Handled, *n* = 15; Hypermotivation [HM]-CUS, *n* = 6; HM-Handled, *n* = 6). **(D)** *CUS* × *timepoint* interaction: *F*_(1, 11)_ = 5.56, *p* = 0.038. Change in CORT levels 15 min after footshock between pre- and late post-CUS tests presented as % of baseline of the total cohort (Total, **G**) and each subgroup (**H–K**). **(G)** *CUS*: *F*_(1, 53)_ = 4.86, ^*^p = 0.032 (in islet). **(J)** *CUS*: *F*_(1, 18)_ = 5.69, ^*^p = 0.028 (in islet). **(K)** *CUS* × *timepoint* interaction: *F*_(1, 10)_ = 5.03, *p* = 0.049. Data are shown as mean + SEM. ^*^p < 0.05 between relevant conditions **(D,K)** or between subgroups **(G,J)**.

In Experiment 2, we aimed to determine whether the four subgroup classifications established in Experiment 1 could predict distinct responses to 4-week CUS (Figure 3A). Another group of experimentally naïve rats was trained to press an active lever with the PR schedule for three sessions in an identical manner to Experiment 1. According to the criteria established in Experiment 1, this second group was successfully divided into the four subgroups as defined above (Supplementary Figure 4). In brief, animals with <1.5 log-transformed active lever presses in the first PR session were classified as the Low Motivation subgroup (Cluster I, *n* =27, 32.1% of all animals) and those with >2 comprised the Hypermotivation subgroup (Cluster IV, *n* =12, 14.3%). Among the remainders, those with lever press change ratios that were greater or lesser than −0.2 between the first and third sessions were classified as the QL (Cluster II, *n* =13, 15.5%) or SL (Cluster III, *n* =32, 38.1%) subgroups, respectively.

Each subgroup was then divided into control and CUS-exposed (stressed) groups. After CUS, we compared the serum CORT levels at rest (Figures 3B–F) and 15 min after a footshock (Figures 3G–K) between control and stressed rats in each subgroup. The pre-CUS CORT at rest did not differ among the four subgroups (data not shown). In the total cohort, CUS had no effect on CORT at the early (CUS Day 25) or late post-CUS time point (post-CUS Day 27; Figure 3B). However, when they were classified into subgroups, there was a significant difference between early and late post-CUS in the stressed QL subgroup [*F*_(1, 11)_ = 19.85; Figure 3D].

CUS reduced the sensitivity to the acute stressor based on the CORT increase in the total cohort (Figure 3G), SL (Figure 3J), and Hypermotivation (Figure 3K) subgroups. In the Hypermotivation subgroup, a significant difference in footshock-induced CORT between the two tests (pre- and late post-CUS) was observed only in handled animals [*F*_(1, 10)_ = 9.83].

We assessed the effects of CUS on body weight gain during the CUS and post-CUS periods (Supplementary Figure 5). CUS altered body weight in the total group and all subgroups except for the Hypermotivation subgroup during the CUS period. In the total cohort, the effects of CUS on body weight appeared after 1 week and lasted until the end of the study. CUS had significant effects on the body weight on all days [*F*s_(1, 738)_ ≥ 7.18), except on CUS Day 1 (*F* < 1). In the Low Motivation subgroup, significant effects were found on all days [*F*s_(1, 225)_ ≥ 4.44], except on CUS Day 1 and post-CUS Day 22 (*F*s ≤ 3.85). In the QL and SL subgroups, the effects were significant on all days [*F*s_(1, 99)_ ≥ 8.99 and *F*s_(1, 270)_ ≥ 6.00, respectively], except CUS Days 1 and 8 (*F*s ≤ 3.23). However, in the Hypermotivation subgroup, the weight change was significant only on post-CUS Day 1 [*F*_(1, 90)_ = 5.53].

### Distinct behavioral reactions to a novel environment after CUS

**Figure 4:**
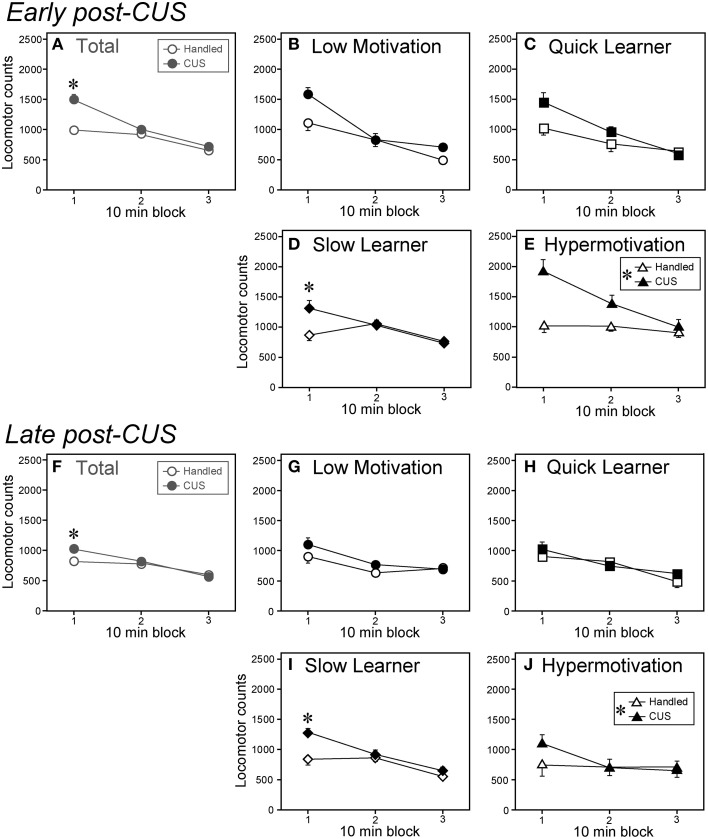
**Chronic unpredictable stress differentially affected locomotor reaction to a novel environment among subgroups. (A–E)** Locomotor counts of CUS-exposed (filled symbols) and handled (open symbols) animals in a NE at Day 3 post-CUS (early post-CUS period). **(F–J)** Locomotor counts in a NE on Day 28 post-CUS (late post-CUS period). Data are shown as mean ± SEM for the total cohort (Total, **A,F**) and each subgroup (**B–E** and **G–J;** Low Motivation, circle; Quick Learner, square; Slow Learner, lozenge; Hypermotivation, triangle. Low Motivation [LM]-CUS, *n* = 15; LM-Handled, *n* = 12; Quick Learner [QL]-CUS, *n* = 6; QL-Handled, *n* = 7; Slow Learner [SL]-CUS, *n* = 17; SL-Handled, *n* = 15; Hypermotivation [HM]-CUS, *n* = 6; HM-Handled, *n* = 6). *CUS* × *time-block* interaction were *F*_(2, 164)_ = 14.56, *p* < 0.0001 **(A,F)** and *F*_(2, 60)_ = 9.38, *p* = 0.0003 **(D,I)**. **(E,J)** *CUS*: *F*_(1, 10)_ = 7.33, ^*^p = 0.022 (in islet). ^*^p < 0.05, significant simple-main effect of *CUS* in a 10-min block or between handled and CUS.

In Experiment 2, we examined the NE-induced behavior on post-CUS Days 3 and 28. Overall, on both the early and late post-CUS days, the stressed animals exhibited enhanced novelty-induced locomotor activity (Figures 4A,F) compared with controls. The CUS effect on locomotor activity was significant only in the first 10-min blocks on both test days [*F*_(1, 246)_ = 48.74]. According to the subgroup analyses, CUS had effects on novelty-induced hyperlocomotion in the SL (Figures 4D,I) and Hypermotivation (Figures 4E,J) subgroups. In the SL subgroup, CUS had significant effects on locomotion only in the first 10-min blocks on both test days [*F*_(1, 90)_ = 22.10: Figures 4D,I). In the Hypermotivation subgroup, CUS had a significant main effect (*p* < 0.05; Figures 4E,J) but without significant interactions. In the Low Motivation (Figures 4B,G) and QL subgroups (Figures 4E,J), we found no main effects of CUS or interactions (*F*s ≤ 3.75).

### Distinct short- and long-term effects of CUS on appetitive instrumental performance

**Figure 5:**
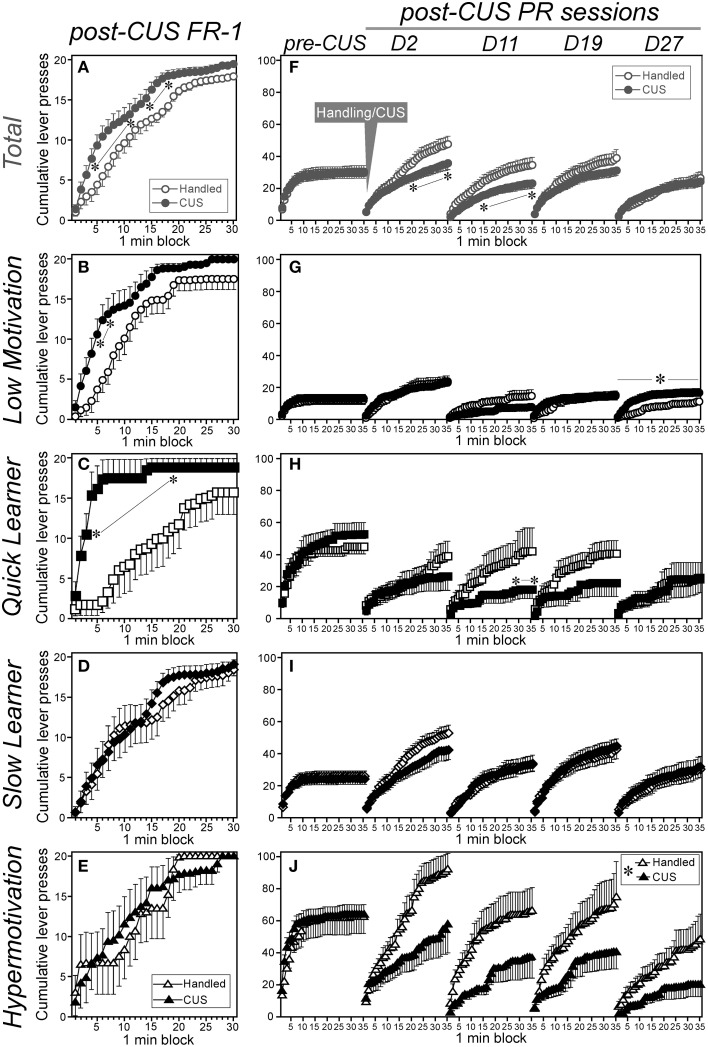
**Chronic unpredictable stress differentially affected constant low-cost or progressive ratio instrumental performance among subgroups**. Cumulative saccharin-reinforced lever presses with the FR-1 (**A–E**; on the day following the last CUS session, left panels) or PR schedule (**F–J**; over time during the pre- and post-CUS periods, right panels) of CUS-exposed (filled symbols) and handled (open symbols) animals in the total cohort **(A,F)** and each subgroup (**B,G**: Low Motivation, circle; **C,H**: Quick Learner, square; **D,I**: Slow Learner, rhombus; **E,J**: Hypermotivation, triangle). Data are shown as mean ± SEM (Low Motivation [LM]-CUS, *n* = 15; LM-Handled, *n* = 12; Quick Learner [QL]-CUS, *n* = 6; QL-Handled, *n* = 7; Slow Learner [SL]-CUS, *n* = 17; SL-Handled, *n* = 15; Hypermotivation [HM]-CUS, *n* = 6; HM-Handled, *n* = 6). *CUS* × *time-block* interaction were: **(A)** *F*_(29, 2378)_ = 1.79, *p* = 0.0060, **(B)** *F*_(29, 725)_ = 1.76, *p* = 0.0083, **(C)** *F*_(29, 319)_ = 3.49, *p* < 0.0001, **(F)** *F*_(136, 11, 152)_ = 1.59, *p* < 0.0001, **(G)** *F*_(4, 100)_ = 6.04, *p* = 0.041, **(H**) *F*_(136, 1496)_ = 1.42, *p* = 0.0017, and **(J)** *F*_(34, 340)_ = 3.69, ^*^p < 0.0001 (in islet). ^*^p < 0.05, significant simple-main effect of *CUS* in a 1-min block **(A–C,F,H)** or in the day **(G)**, or between handled and CUS **(J)**.

In Experiment 2, we examined the instrumental lever pressing performance during the post-CUS period without water or food restrictions. On the day after the last CUS session, performance in low-cost instrumental learning (FR-1 schedule) was assessed (Figures 5A–E). Next, the, PR instrumental performance was assessed once each week (Figures 5F–J). In the FR-1 session, active lever pressing by the total cohort was accelerated by CUS [time-blocks 4-11 and 14-48, *F*s_(1, 2460)_ ≥ 4.38; Figure 5A]. Significantly enhanced lever pressing was also found in the Low Motivation [time-blocks 5-7, *F*s_(1, 750)_ ≥ 5.21; Figure 5B], and QL subgroups [time-blocks 3–19, *F*s_(1, 330)_ ≥ 4.16; Figure 5C) but not in the SL and Hypermotivation subgroups (Figures 5D,E).

By contrast, CUS reduced the instrumental performance with the PR schedule in the total cohort both immediately after the CUS cessation [Day 2, time-blocks 21-35, *F*s_(1,14,350)_ ≥ 4.91] and on Day 11 (time-blocks 15-35, *F*s ≥ 4.12) of the post-CUS period (Figure 5F). Reductions in the PR performance occurred in the QL on Day 11 [time-blocks 29-35, *F*s_(1, 1925)_ ≥ 4.05; Figure 5H] and Hypermotivation [time-blocks 10-35, *F*s_(1, 350)_ ≥ 3.92; Figure 5J] subgroups regardless of the test days. However, the Low Motivation subgroup did not exhibit significant changes in PR performance until about 3 weeks post-CUS and on Day 27, the PR performance was significantly enhanced in stressed animals [*F*s_(1, 125)_ = 4.54: Figure 5G]. In the SL subgroup, the main effects of CUS and the CUS-related interactions were not significant (Figure 5I).

All of these distinctive behavioral characteristics of the four subgroups are summarized in Supplementary Table 2.

## Discussion

In this study, we classified naïve Sprague-Dawley rats into four subgroups by taking advantage of an instrumental learning with a PR schedule where the motivation for reward and the process of the instrumental learning could be assessed. The animals in each subgroup exhibited characteristic cognitive and behavioral traits at baseline as well as distinct behavioral responses to stress (CUS), although some of them are not involved as essential criteria for diagnosing MDD. Given the potential translational value of these results, we discuss possible relationships between the pre-CUS traits in rats and premorbid temperament in humans as well as between the post-CUS phenotypes in rats and MDD symptoms in humans.

### Practical application of subgroup classification based on PR performance for identifying individual differences at the pre-stress stage

We classified the animals into behavioral subgroups (Low Motivation, QL, SL, and Hypermotivation) based on behavioral performance followed by hierarchical cluster analysis (Figures 1B–E, Supplementary Figure 1). In Experiment 1, we found that individual differences in PR performance exceed those in FR performance (Supplementary Figure 2). PR-based classification was also sensitive to outcome devaluation (Figure 2A), errors made during set-shifting (Figure 2B), and cocaine-induced behaviors (Supplementary Figure 3). These data suggest that many behavioral and cognitive variables may be functionally related to each other and they can be segregated into small subgroups at pre-stress stage by PR performance.

### Cognitive and behavioral traits of each subgroup in the pre-stress state

Low Motivation animals were designated because of the least active lever presses during the first PR session, no further changes in the number of lever presses during the subsequent two sessions (Figures 1D–F), and significantly fewer active lever presses during the progression of the intermittent reinforcement schedule (Supplementary Figure 2A). Loss of motivation is one of the representative MDD symptoms. A similar phenotype is observed in rats with dopamine depletion in the nucleus accumbens, which is essential for motivation (Salamone and Correa, 2012). Although amotivation at premorbid state may appear odd, a recent human study suggests that adolescents with lower scores in the power and achievement motives may be vulnerable to MDD (Fuhr et al., 2014).

QL and SL subgroups were not distinguishable based on their active lever press rates during the first PR session (Figure 1D) and most of the FR sessions (Supplementary Figure 2A) but they could be differentiated by their change ratios (Figures 1E,F). The QL subgroup members also exhibited a different sensitivity to outcome devaluation compared with the SL subgroup (Figure 2A), thereby suggesting that the habitual process of instrumental learning was facilitated better in the QL subgroup than the SL subgroup (Yin and Knowlton, 2006).

We also found the lesser errors were made by the QL subgroup compared with by the SL subgroups in set-shifting (Figure 2B). Rapid adaptation to a newly introduced discrimination rule (reflected by fewer perseverative errors) appeared to be paralleled by rapid learning in this subgroup. QL was designated because of this general rapid learning property. Alternatively, it is possible that they are too impulsive to make action, which may result in a quick but premature learning. This idea is supported by the facilitation of habitual drug seeking by an impulsive predisposition (Everitt et al., 2008). By contrast, the SL subgroup exhibited the opposite trait in learning, namely greater cognitive persistence or slow learning. This subgroup comprised 16% of total, which appears to be excessively high to define a learning disability, so this subgroup may tend to be more prudent or perserverative (=less impulsive), and they may require numerous trials and errors to process new information or make decisions. In consistent with this, rats characterized at risk averse make more perseverative errors on set shifting (Shimp et al., 2015).

Finally, the Hypermotivation subgroup was designated because of the distinguished higher number of active lever presses than the others in the first PR session (Figures 1D,F) and a notable increase in active lever presses with the intermittent FR schedule (Supplementary Figure 2). During PR training, the reward provided was not accompanied by any caloric value or water/food deprivation, so the Hypermotivation subgroup was seeking excessively for a sweetness sensation and not for caloric value. Escalated novelty/sensation seeking is a characteristic feature of manic states and attention-deficit/hyperactive disorder (Hegerl et al., 2010), as well as addiction-prone individuals (Steinberg, 2004; Cservenka et al., 2013). This pre-stress trait may correspond to hyperthymic temperament, which is one of the premorbid traits of bipolar disorder-like phenotypes in humans. Furthermore, this subgroup specifically exhibited an enhanced response to acute cocaine, another hallmark of the mania-like phenotype (Roybal et al., 2007, but see Hegerl et al., 2010; Supplementary Figure 3B).

### Distinct effects of CUS on each subgroup

#### PR performance

CUS induced loss of motivation to total cohort as shown in Figure 5F. The subsequent subgroup analyses revealed that this was mainly attributable to the Hypermotivation but partly to the QL subgroups (Figures 5H,J). In the Hypermotivation subgroup, although their motivation was still at the normal level from an objective viewpoint, they may have subjectively felt demotivated compared with their previous levels.

In case of the QL subgroup, the effect of CUS was transient and less robust effect compared with the Hypermotivation subgroup (Figure 5J). Alternatively, the natural rational process involved in reducing the active lever pressing may have been augmented in the stressed QL animals, which exhibited rapid adaptive learning as a pre-stress trait.

The SL subgroup displayed no effects of CUS throughout the entire post-CUS period in any respect, thereby implicating the most resistant to stress-induced demotivation among all.

Finally, the stressed Low Motivation animals began to press the active lever pressing more frequently than the controls following CUS from around post-CUS Day 27. In theory, active lever pressing should decrease gradually with the PR sessions, so this action appears to be unusual. However, because CUS favors habitual actions rather than flexible goal-directed actions (Dias-Ferreira et al., 2009), it is possible that an old habitual learning overwhelmed a recent flexible behavioral modification in a late-developing manner. Behavioral/cognitive inflexibility (or inadequate decision-making), which may result in extremely biased thoughts such as suicidal ideation, is one of the major hallmarks of MDD (Miranda et al., 2013). Further studies are necessary to address this issue.

#### Body weight

In terms of body weight, CUS selectively affected the Low Motivation subgroup, thereby reflecting stress-induced appetite or metabolic suppression, whereas the Hypermotivation subgroup was almost resistant (Supplementary Figure 5).

#### CORT

Stress-induced elevation of CORT is considered to reflect the enhanced activity of hypothalamic-pituitary-adrenal (HPA) axis in MDD, although its functional significance is still debated (Kunzel et al., 2003; Lamers et al., 2013). At early post-CUS, CUS displayed its effect on CORT only in the QL subgroup in a temporal manner (Figure 3D). Given that habituation of the HPA-axis to repeated stress can function by attenuating the behavioral response to stress, such as general anxiety (Uchida et al., 2008), the QL subgroup may be resistant to CUS.

Meanwhile, CUS suppressed the acute footshock-induced increase in CORT in the SL and Hypermotivation subgroups (Figures 3J–K). However, the habituation to acute stress in these subgroups may lead to context- or object-specific anxiety, as reported in posttraumatic stress disorder (Santa Ana et al., 2006).

#### Other behavioral indices

CUS enhanced locomotor response in the NE in the SL and Hypermotivation subgroups (Figure 4). This behavioral augmentation may be functionally linked to the aforementioned blunted HPA-axis response to acute stress in these two subgroups, although the precise mechanism underlying it remains elusive.

Overall, our findings strongly suggest that distinct pre-CUS characteristics represented rather “traits” that were attributable to long-term functional interactions of various behavioral/cognitive properties, than merely “states” that reflected temporal biological or neural situations of each individual.

### Potential advantages and concerns

As mentioned above, the limited effectiveness of antidepressants and the difficulty in predicting their effectiveness before medication are in part due to the current homogenous model of MDD. Thus, if the relationships between each subgroup with the response to different types of antidepressants can be clarified, more effective custom-made therapies could be formulated for each MDD individual.

It is still unclear whether the observed stress-response heterogeneity is based on genetic or epigenetic factors. In this study, we pooled two experimental sessions because it was difficult to test many animals simultaneously. Although all four subgroups were equally represented in both sessions, any differences in external factors, such as hierarchies among cage mates, transport conditions from a vendor, or any pre- or post-natal stress, may have been involved epigenetically (Fitzpatrick et al., 2013).

Another potential issue is the specificity of the CUS-induced phenotypes to MDD. Similar to MDD, onset or relapse is triggered by stress in many psychiatric disorders. In addition, many stress-induced symptoms such as depressive mood and loss of motivation are not specific to MDD. Therefore, it is possible that CUS may evoke phenotypes that are related to other mental disorders, particularly bipolar disorders. The identification of the hypermotivation subgroup as a pre-stress trait may support this possibility.

Finally, although this study could open another door to further understanding of MDD and its therapeutic approaches, as animal model of MDD it definitely needs to be validated with the response to antidepressants. Following studies employing antidepressants will be necessary.

In summary, we classified a group of experimentally naïve rats into four subgroups: Low Motivation, Quick Learner (QL), Slow Learner (SL), and Hypermotivation. We then clarified the effects of CUS on each subgroup. The Low Motivation subgroup exhibited lower motivation for the reward before CUS. After CUS, this subgroup exhibited weight loss and a late-developing paradoxical enhancement in PR performance which may be related to inappropriate decision-making in human MDD. The OL subgroup switched cognitive strategies rapidly at pre-CUS, which may reflect impulsivity. After CUS, this subgroup exhibited a transient loss of motivation (or rapid adaptive behavior) and the habituation of the HPA-axis response to repeated stress which may reflect resilience to general anxiety. The SL subgroup displayed prudency or persistency with respect to the initially acquired strategies at pre-CUS. After CUS, this subgroup exhibited resistance to demotivation and a suppressed HPA axis response to acute stress, so they may be prone to context- or object-specific anxiety. Finally, the Hypermotivation subgroup exhibited enhanced reward and sensation seeking as pre-CUS traits, which may correspond to hyperthymia/hyperactivity in humans. After CUS, this subgroup exhibited resistance to weight loss, habituated HPA response to an acute stress, and a long-lasting amotivation. It was notable that some of the CUS-induced phenotypes appeared to be anti-MDD-like rather than pro-MDD. The Low Motivation subgroup is probably the most vulnerable, whereas the QL and SL subgroups are relatively resistant to stress. Overall, distinct causal relationships between pre-stress traits and post-stress phenotypes were identified in each subgroup. Thus, the consequences of stress may be predicted before stress exposure by identifying the pre-stress traits of each individual (summarized in Table 1).

**Table 1:** **A summary of causal relationships between putative pre-stress traits in humans that may correspond to pre-CUS traits in rats and putative post-stress MDD-related phenotypes that may correspond to post-CUS phenotypes in rats**.

**Subgroup**	**Putative pre-stress traits in humans corresponding to pre-CUS traits in rats**	**Putative post-stress symptoms in human MDD corresponding to post-CUS phenotypes in rats**
Low motivation	Low motivation	Weight loss/Inflexible decision making
Quick learner	Impulsivity	Transient loss of motivation *or enhanced adaptive behavior/Resistance to general anxiety*
Slow learner	Prudency or Perseveration	*Resistance to amotivation*/Context- or object-specific anxiety
Hypermotivation	*Hyperthymia or Hyperactivity*	Long-lasting amotivation/Context- or object-specific anxiety/*Resistance to weight loss*

*Regular, pro-MDD-like, italic, anti-MDD-like traits or phenotypes*.

## Author contributions

YI, SK, YM, and ST designed the experiments; YI, SK, ZL and HN performed all experiments; YI and ST analyzed data; YI, YM and ST wrote the manuscript.

### Conflict of interest statement

We have received funding from two commercial sources, Eli Lilly and Takeda. However, we declare that they do not alter our adherence to all Frontiers policies on sharing data and materials. The authors declare that the research was conducted in the absence of any commercial or financial relationships that could be construed as a potential conflict of interest.

## Abbreviations

ADHD: attention-deficit/hyperactive disorder
ANOVA: analyses of variance
CORT: serum corticosterone
CUS: chronic unpredictable stress
FR: fixed ratio
HPA: hypothalamic-pituitary-adrenal
ITI: inter-trial interval condition
MDD: major depressive disorder
NE: novel environment
PR: progressive ratio.
