# Supplementary material for: Pre-stress performance in an instrumental training predicts post-stress behavioral alterations in chronically stressed rats
Source: Front Behav Neurosci. 2015 May 13;9:119. doi: 10.3389/fnbeh.2015.00119 (PMC4429589; doi:10.3389/fnbeh.2015.00119)
Supplement: Supplementary file 1 [file DataSheet1.DOCX]

***Supplementary Material***

**Pre-stress performance in an instrumental training predicts post-stress behavioral alterations in chronically stressed rats**

**Yoshio Iguchi^1^, Sakurako Kosugi^1^, Ziqiao Lin^1^, Hiromi Nishikawa^1^, Yoshio Minabe^1,2^, and Shigenobu Toda^1,2^***

^1^*Department of Psychiatry & Neurobiology, Kanazawa University School of Medicine, Kanazawa, Ishikawa, Japan*

^2^*Research Center for Child Mental Development, Kanazawa University, Kanazawa, Ishikawa, Japan*

*** Correspondence:** Shigenobu Toda, M.D. Ph.D. Department of Psychiatry and Neurobiology, Kanazawa University School of Medicine, 13-1 Takara-machi, Kanazawa, Ishikawa, 920-8641, Japan. Tel: +81-76-265-2308; Fax: +81-76-234-4254; E-mail: todas@med.kanazawa-u.ac.jp

1. **Supplementary Figures and Tables**

## Supplementary Tables


**Supplementary Table 1. Relative size of behavioral characteristics of 4 subgroups classified based on a PR instrumental performance.**

|  | Subgroup (cluster) | | | |
| --- | --- | --- | --- | --- |
| *Behavioral variables* | **Low Motivation (I)** | **Quick Learner (II)** | **Slow Learner (III)** | **Hyper-motivation (IV)** |
| Lever presses in the first session of PR training |  | **+** | **+** | **++** |
| Reduction in lever presses from the first to third PR sessions |  |  | **++** | **+** |
| Fixed-ratio 1/2 instrumental performance w/o deprivation |  | **+** |  | **++** |
| Fixed-ratio 5 instrumental performance w/o deprivation |  | **+** | **+** | **++** |
| Sensitivity to outcome devaluation | **+** |  | **+** |  |
| Errors committed in set-shifting |  |  | **+** |  |
| Acute cocaine-induced hyperlocomotion |  |  |  | **+** |

**Supplementary Table 2. Relative effect sizes of CUS on each behavioral variable in 4 subgroups.**

|  | Subgroup (cluster) | | | |
| --- | --- | --- | --- | --- |
| *Behavioral variables* | **Low Motivation (I)** | **Quick Learner**  **(II)** | **Slow Learner (III)** | **Hyper-motivation (IV)** |
| Resting state CORT at early post-CUS |  | **+** |  |  |
| Long-term habituation in CORT response to acute stress |  |  | **+** | **+** |
| Suppression of body-weight increase during CUS | **+++** | **++** | **++** | **+** |
| Suppression of body-weight increase during CUS-free phase | **+** | **+** | **+** |  |
| Novelty-induced hyper locomotion at early post-CUS |  |  | **+** | **+** |
| Long-term novelty-induced hyper locomotion |  |  | **+** | **+** |
| Enhancement in low-cost (FR-1) instrumental performance at early post-CUS | **+** | **+** |  |  |
| Reduction in PR instrumental performance at early post-CUS |  |  |  | **+** |
| Long-term reduction in PR instrumental performance |  |  |  | **+** |
| Late-developing and transient reduction in PR instrumental performance |  | **+** |  |  |
| Late-developing escalation in PR instrumental performance | **+** |  |  |  |

*Notes:* CORT = serum corticosterone concentration; FR = fixed ratio; PR = progressive ratio

## Supplementary Figures





**Supplementary Figure 1. Cluster dendrogram for a group of naïve male Sprague–Dawley rats in Experiment 1.** Hierarchical clustering was performed on the Euclidean square distances between data plotted on a 2-factorial surface (Figure 1C). *x*-axis: number of lever presses during the first PR session normalized by a common logarithm (log) transformation; *y*-axis: change ratios in lever presses from first to third PR sessions. Based on the data, animals (*n* = 79) were classified into 4 subgroups (dotted line): Cluster I, *n* = 17 (21.5% of the whole animals); Cluster II, *n* = 36 (45.6%); Cluster III, *n* = 13 (16.5%); Cluster IV, *n* = 13 (16.5%).


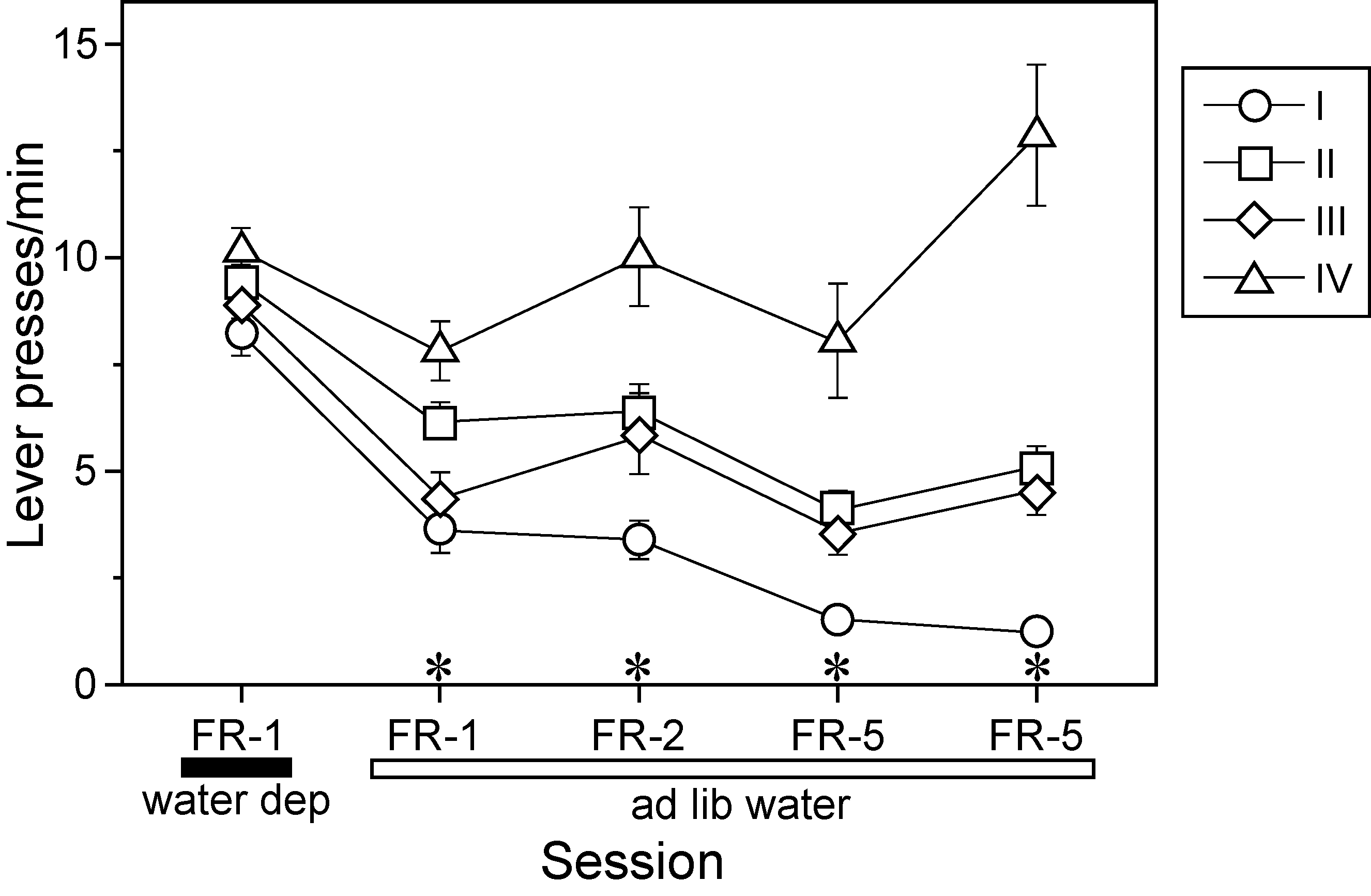


**Supplementary Figure 2. Presses on active lever during FR instrumental training sessions (before the 7-session PR training) in Experiment 1.** Data are shown as subgroup mean ± *SEM* (Cluster I, *n* = 17; II, *n* = 36; III, *n* = 13; IV, *n* = 13). In the first FR-1 session, animals were water deprived in their home cages for 23.5 h/day (black bar). *Subgroup*: *F*_(3, 75)_ = 2.48, *p* = 0.067. For the subsequent 4 sessions (days), they had *ad libitum* access to water (white bar). *Subgroup* × *session* interaction: *F*_(9, 225)_ = 6.03, *p* < 0.011; ^*^*p* < 0.05, significant simple-main effects of *subgroup* in a session.

**
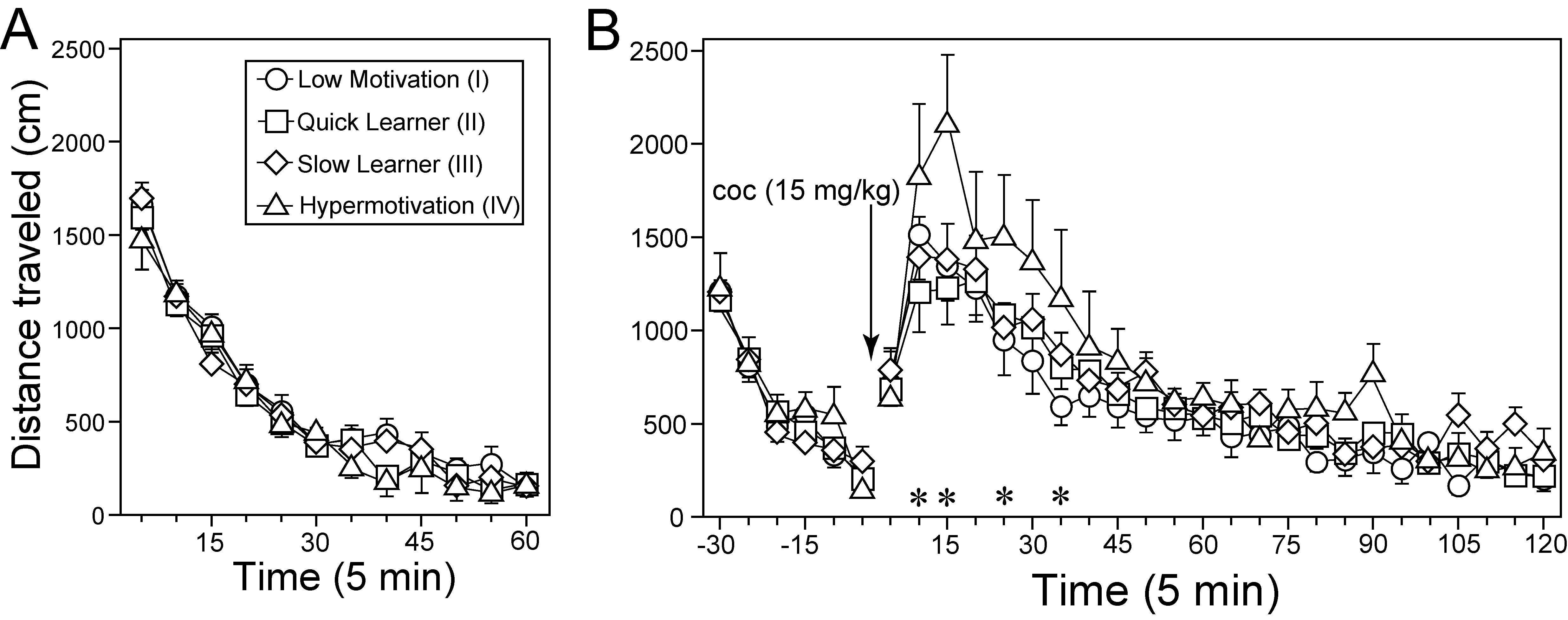
**

**Supplementary Figure 3. Distance traveled in a novel environment (A) and before and after acute administration of cocaine (B) in Experiment 1.** Data are presented as subgroup mean ± *SEM* (Cluster I, *n* = 10; II, *n* = 16; III, *n* = 11; IV, *n* = 5). **(A)**. *Subgroup* main effect before cocaine injection: *F*_(3, 38)_ < 1. *Subgroup* × *time-block* interaction before cocaine injection: *F*_(33, 418)_ < 1. **(B)** *Subgroup* main effect before cocaine injection: *F*_(3, 38)_ < 1. *Subgroup* × *time-block* interaction before cocaine injection: *F*_(15, 190)_ = 1.47, *p* =0 .12. *Subgroup* × *time-block* interaction after cocaine injection: *F*_(69, 874)_ = 1.27, *p* = 0.076; ^*^*p* < 0.05, significant simple-main effects of *subgroup* in a time-block.


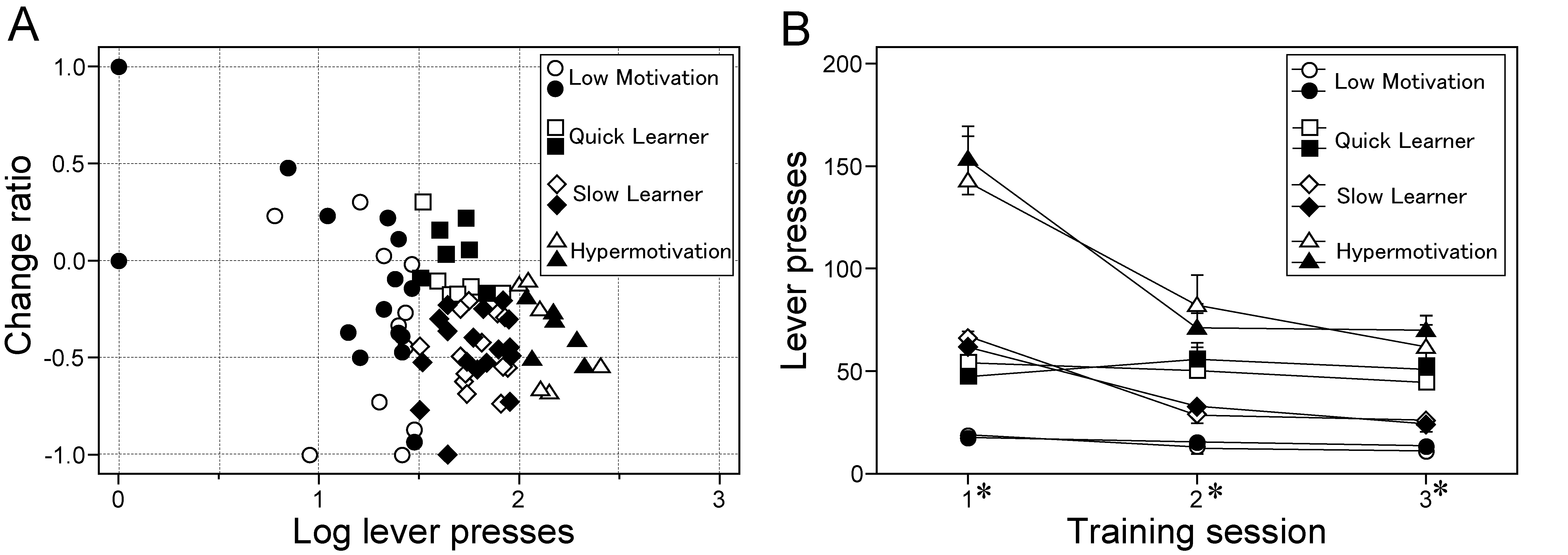


**Supplementary Figure 4. Subgroup classification based on 3 PR sessions in Experiment 2.** **(A)** Performances of individual rats in 3 PR training sessions summarized on a 2-factorial graph. *x*-axis: active lever press number on the first PR session (common logarithm-transformed); *y*-axis: change ratios of lever presses from first to third sessions. Rats were classified into 4 subgroups by using the criteria established in Experiment 1. Filled symbols represent CUS-exposed animals, open symbols represent handled animals. **(B)** Active lever presses by each subgroup (Low Motivation, LM, circle; Quick Learner, QL, square; Slow Learner, SL, lozenge; Hypermotivation, HM, triangle) during the 3 PR training sessions. CUS-exposed animals (filled symbols) and Handled controls (open symbols) were separately displayed in each subgroup. Data presented as subgroup mean ± *SEM* (LM-CUS, *n* = 15; LM-Handled, *n* = 12; QL-CUS, *n* = 6; QL-Handled, *n* = 7; SL-CUS, *n* = 17; SL-Handled, *n* = 15; HM-CUS, *n* = 6; HM-Handled, *n* = 6)*. Subgroup* × *session* interaction: *F*_(6, 152)_ = 31.38, *p* < 0.0001; ^*^*p* < 0.05, significant simple-main effects of *subgroup* in a session. In the first session, all between-subgroup differences reached significant level (*t*s_(228)_ ≥ 4.92) except between QL and SL subgroups (*t* = 1.98). In the third session, all between-subgroup differences were significant (*t*s ≥ 2.36) except between QL and HM subgroups (*t* = 2.18).


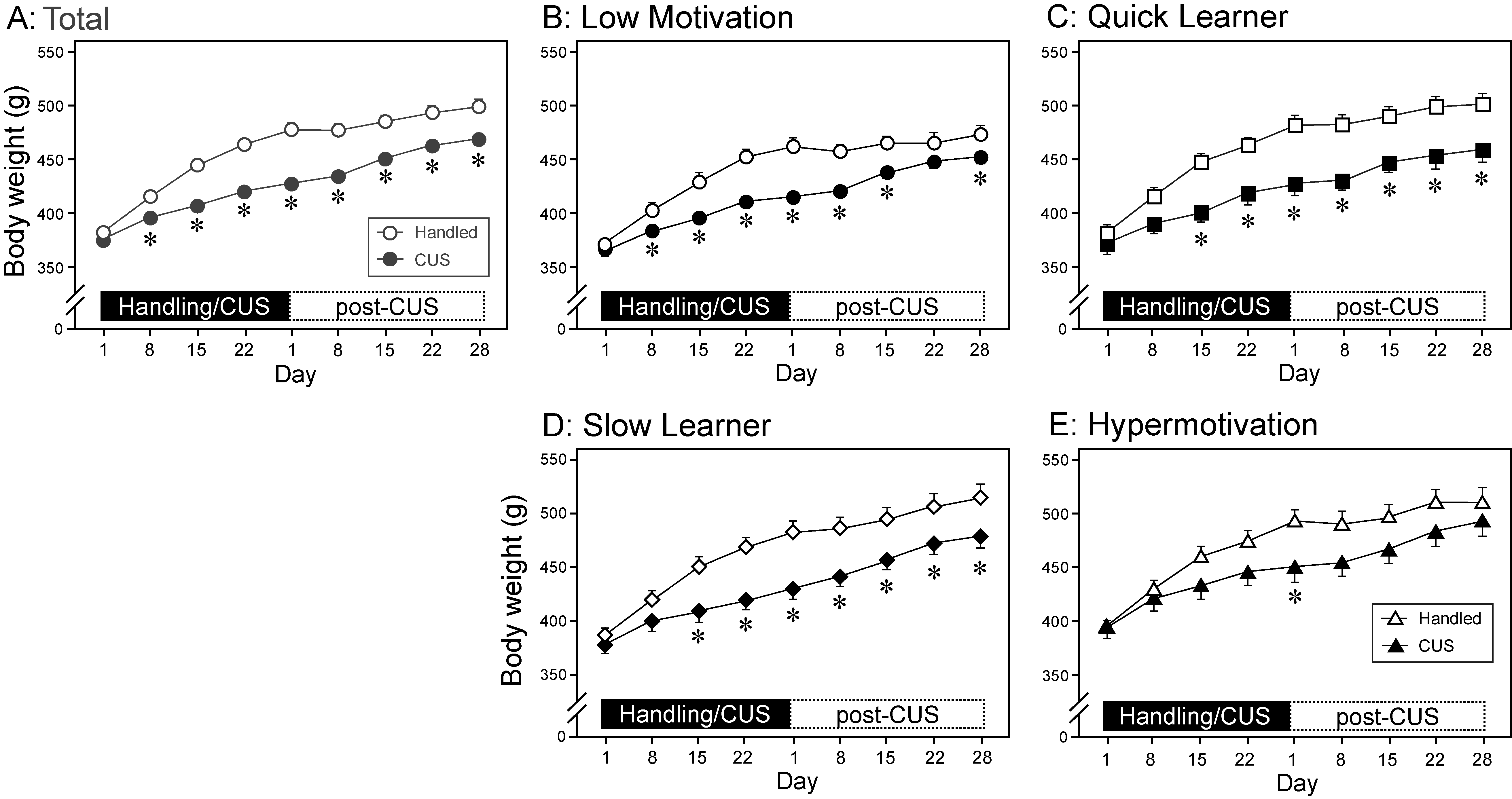


**Supplemental Figure 5. Chronic unpredictable stress differentially suppressed body weight gain among subgroups**. Body weights of CUS-exposed animals (filled symbols) and handled controls (open symbols) in each subgroup on the 28-day CUS (/handling) period and the following 28-day post-CUS period. Data are shown as mean ± *SEM* (Low motivation [LM]-CUS, *n* = 15; LM-Handled, *n* = 12; Quick Learner [QL]-CUS, *n* = 6; QL-Handled, *n* = 7; Slow Learner [SL]-CUS, *n* = 17; SL-Handled, *n* = 15; Hypermotivation [HM]-CUS, *n* = 6; HM-Handled, *n* = 6) **(A)** Total cohort, *CUS* × *Day* interaction: *F*_(8, 656)_ = 37.33, *p* < 0.0001. **(B)** LM, *CUS* × *Day* interaction: *F*_(8, 200)_ = 16.72, *p* < 0.0001. **(C)** QL, *CUS* × *Day* interaction: *F*_(8, 88)_ = 10.11, *p* < 0.0001. **(D)** SL, *CUS* × *Day* interaction: *F*_(8, 240)_ = 13.00, *p* < 0.0001. **(E)** HM, *CUS* × *Day* interaction: *F*_(8, 80)_ = 5.50, *p* < 0.0001. ^*^*p* < .05, significant simple-main effects of *CUS* in a day.
